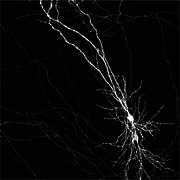# Pyramidal neurons of CA1 neuron in organotypic slice

**DOI:** 10.1002/alz.084527

**Published:** 2025-01-03

**Authors:** Avishek Roy, Severine Deforges, Christophe Mulle

**Affiliations:** ^1^ Interdisciplinary Institute for Neuroscience (UMR 5297), University of Bordeaux, Bordeaux, Gironde France

## Abstract

This is a maximal intensity projection of CA1 pyramidal cell transfected with plasmid with the reporter GFP using single cell electroporation technique. In this particular case the organotypic slices were prepared from p5‐7 pups in a tissue chopper (McIlwain). And maintained in MEM bases media with added glutamax with a change in 2 alternative dyas at 37°C and 5% CO_2_ for 4 days in‐vitro (DIV) before electroporating with a glass pipette of 7‐10mΩ resistance by applying 4 square pulses of ‐ve voltage of −2.5 V, for 25 ms duration at 1 Hz frequency. Then maintained in the same medium for one four more days before fixing them in PFA(4‰ v/v) and amplified with anti‐GFP antibody (ab290, 1:1000 dil) and imaged in SP2 confocal microscope (Leica) for z‐stack.